# Exploring the Interplay between Stress Perception and Approach-Avoidance Achievement Goal Orientation among Rural Students in College: A Longitudinal Study in China

**DOI:** 10.3390/bs14090774

**Published:** 2024-09-03

**Authors:** Yunfeng Luo, Wenjuan Gao, Xinqiao Liu

**Affiliations:** 1 School of Public Administration, University of Electronic Science and Technology of China, Chengdu 611731, China; 2 School of Public Administration, Beihang University, Beijing 100191, China; wjgao@buaa.edu.cn; 3 School of Education, Tianjin University, Tianjin 300350, China

**Keywords:** stress perception, approach goals, avoidance goals, rural students, longitudinal study, cross-lagged models

## Abstract

The objective of this study was to examine the relationship between stress perception and achievement goals among rural college students in China. Using a two-wave random intercept cross-lagged model, this study analyzed data from a longitudinal survey conducted at 15 public universities in Beijing. First, there was a significant decline in stress levels among Chinese rural students from their junior to senior years. Second, the achievement goal orientations of these students remained relatively stable during the period. Third, while no significant association was found between approach goals and stress perception, a clear positive reciprocal relationship emerged between avoidance goals and stress perception. These results suggest that educators should focus on alleviating the stress experienced by rural college students through academic, employment, and interpersonal support to break the vicious cycle of stress perception and avoidance goal orientation. Future research should further explore the influence of diverse group characteristics and emotional states on these outcomes.

## 1. Introduction

Stress has emerged as a prevalent psychological issue among student populations, particularly in higher education [1,2]. A survey of 2831 college students from Germany and Luxembourg indicated that 45% of respondents experienced elevated stress levels [3]. Academic stress is the primary stressor for college students [4], as they face a more demanding curriculum, rigorous evaluation standards, and intense academic competition [5]. The COVID-19 pandemic has exacerbated academic stress, largely due to the implementation of physical distancing measures. The abrupt shift from traditional in-person classes to remote online learning inevitably led to a decline in students’ learning motivation and heightened the pressure of independent study [6]. Employment stress is another critical issue that cannot be overlooked [7]. The pandemic has significantly impacted global economic activities and employment opportunities [8,9], heightening concerns and anxieties about future careers among college students. For instance, a survey of 2627 final-year college students in China found that approximately 53.3% perceived the current employment situation as severe [10]. Furthermore, interpersonal stress remains a significant concern for college students [11]. College life represents a crucial transition period from a familial environment to independent living, requiring students to form new interpersonal relationships and adapt to diverse social settings. Due to individual differences, some students may struggle with interpersonal interactions, resulting in feelings of loneliness and helplessness, which contribute to interpersonal stress [12].

Rural students are primarily individuals who have grown up and lived with their parents in rural areas [13]. The Hukou system, a distinctive household registration framework embedded within China’s public administration, categorizes individuals as either urban or rural, with the latter typically receiving fewer social privileges [14,15]. Consequently, rural students face several familial challenges. They often feel a strong sense of responsibility to acknowledge and repay their parents’ years of hard work and frugality by improving their family’s quality of life [16]. Attaining a degree and securing employment are viewed as pathways to fulfilling these responsibilities [17]. As a result, they may experience heightened academic stress, necessitating greater effort to excel in their studies. In terms of employment stress, rural students frequently lack social capital [18], which puts them at a disadvantage in the highly competitive job market. Additionally, feelings of inadequacy and inferiority stemming from rural-urban and academic disparities hinder their ability to navigate interpersonal relationships effectively [19,20]. Overall, rural students experience higher levels of stress [21], which can significantly impact their physical and mental health [22], as well as their academic performance [23]. Increased stress has been linked to negative behaviors such as meal skipping and smoking [24], and a higher prevalence of overweight and obesity [25,26]. Stress is also significantly associated with academic self-efficacy [27,28], motivation [29,30], and time management [31,32], all of which have a substantial impact on academic performance. Given these considerations, the psychological challenges faced by rural students warrant particular attention.

Achievement goals significantly influence students’ academic performance, as they represent the underlying purposes for engaging in a task [33]. In the 1970s, scholars categorized achievement goals into two main types: mastery goals and performance goals [34,35]. Mastery goals focus on acquiring knowledge and improving abilities, while performance goals involve demonstrating one’s abilities relative to others in a specific task, emphasizing social comparison and the attainment of recognition and status. Further research by Elliot et al. expanded the concept by introducing a 2 × 2 achievement goal framework, which includes mastery-approach, mastery-avoidance, performance-approach, and performance-avoidance goals [36], This framework was later developed into a 3 × 2 model comprising six goal structures: task-approach, task-avoidance, self-approach, self-avoidance, other-approach, and other-avoidance [37]. These frameworks have been extensively tested in various studies, contributing to the theoretical development of achievement goal literature [38,39,40,41,42].

Elliot and Harackiewicz [43] proposed a framework categorizing achievement goal orientation into independent approach and avoidance motivational orientations. This concept builds on McClelland’s theory of achievement motivation [44], which distinguishes between two types of achievement motivation: one focused on avoiding failure and the other on attaining success. Approach goals reflect an individual’s motivation to pursue positive outcomes, such as acquiring new knowledge, achieving good grades, or fostering personal growth. Conversely, avoidance goals are driven by motivations aimed at preventing negative outcomes, such as avoiding failure, criticism, or ignorance. The distinction between approach and avoidance goals has been linked to academic performance and emotional outcomes. Approach goals positively influence students by stimulating intrinsic motivation [45]. When students are motivated by the desire to achieve positive outcomes, they are more likely to enjoy the learning process, which enhances their willingness to invest time and effort in understanding course content. This intrinsic motivation fosters resilience and commitment, helping students maintain a positive attitude when facing learning challenges and continuously seek improvement. Moreover, approach goals are associated with higher levels of self-efficacy, confidence in one’s ability to accomplish specific tasks [46]. In contrast, avoidance goals are often linked to extrinsic motivation [47]. This may result in surface learning strategies, such as rote memorization, aiming at coping with exams and avoiding failure. While this approach may yield short-term success, it does not promote deep understanding or long-term retention of knowledge. Students who rely on avoidance strategies may also shy away from challenging tasks due to an excessive fear of failure, limiting their opportunities for learning and personal growth. Avoidance goals may also threaten students’ self-worth [48]. When students’ self-worth is closely tied to academic performance, failures or criticism may lead to feelings of diminished self-worth, prompting avoidance behaviors such as disengagement from learning or school-related activities. Overall, the approach-avoidance distinction, supported by physiological and psychological theories, is more commonly used in classifying achievement goal orientations than the mastery-performance distinction [49].

According to Elliot’s hierarchical model of approach-avoidance motivation [50], avoidance motivation is geared towards survival, while approach motivation is aimed at thriving. This framework indicates that positively evaluated stimuli are inherently associated with an approach orientation, whereas negatively evaluated stimuli correspond with an avoidance orientation. Therefore, stress, as a negative stimulus, should theoretically correlate more directly with avoidance goals [51] rather than with approach goals, leading to the formulation of Hypothesis 1:

**Hypothesis** **1.** *The stress perception of rural students positively predicts their avoidance goals.*

The approach-avoidance model of coping proposed by Susan Roth and Lawrence J. Cohen [52] offers valuable insights into the impact of avoidance and approach goals on stress. The model suggests that both types of goals influence stress levels. When individuals use approach strategies to cope with stress, their cognitive and emotional engagement with the stressor often results in a more comprehensive experience and expression of emotional distress, which can be beneficial. However, focusing too much on threatening stimuli can also heighten distress. Additionally, when individuals cannot change the situation, approach strategies may lead to unproductive worrying [52]. For rural students, engaging in learning for its intrinsic value often brings satisfaction from the learning process itself [53]. This positive emotional experience can alleviate stress [54]. However, approach goals typically require prolonged and unwavering commitment from students. As rural students pursue these goals, they may encounter unforeseen obstacles and challenges, which could potentially increase their stress levels. Taken together, the overall impact of approach goals on stress levels among rural students may be negligible.

When avoidant strategies are employed to cope with stress, they can effectively reduce stress and anxiety by allowing individuals to gradually acknowledge threats [55]. However, avoidant strategies may hinder the ability to take appropriate actions when opportunities to address the threat arise [56]. These strategies can lead to emotional numbness, unwanted intrusions of threatening thoughts, and disruptive avoidance behaviors, which result from conscious or unconscious efforts to suppress distressing cognitions [52]. For rural students, perceptions of stressors are often deeply ingrained and stable, largely stemming from their backgrounds [57]. In the short term, avoidance goals may not effectively alleviate their stress. When rural students set avoidance goals, they are often driven by a fear of failure and its consequences, such as failing tests, losing face among peers, or receiving negative feedback from teachers. This fear can lead to avoidance behaviors among rural students [58], such as disengaging from school-related activities. Such behaviors not only fail to address the underlying issues but may also exacerbate the psychological stress experienced by these students. Therefore, a positive relationship is anticipated between avoidance goals and stress perception among rural students. Accordingly, this study proposed the Hypothesis 2:

**Hypothesis** **2.** *Approach goals do not significantly associate with rural students’ stress perception, whereas avoidance goals positively predict rural students’ stress perception.*

## 2. Materials and Methods

### 2.1. Participants

The data for this study was obtained from a student tracking survey conducted across 15 public universities in Beijing, China [59]. The survey employed a stratified, multi-stage, probability proportional sampling methodology. Firstly, the survey constructed six sampling frames using the student registration database provided by the Beijing Municipal Commission of Education, with samples drawn based on three stages of universities, majors, and students. Secondly, with the cooperation of the Beijing Municipal Commission of Education and student management departments of universities, the selected students were notified. Lastly, surveyors were dispatched to the participating universities to provide on-site guidance during the completion of the questionnaire. The survey included a sample of 655 rural students with a mean age of 22 years. The study involved two phases of data collection: the baseline survey took place in June of the third year, and the follow-up survey occurred from May to July of the fourth year, with a 1-year interval between the two. At baseline, 655 rural students were surveyed to gather initial information on their stress levels and achievement goals during their junior year of college. This cohort of students was surveyed again during their senior year, with 542 rural students providing relevant information in the follow-up survey, resulting in an attrition rate of 17.25%. There were no significant differences between the participants at baseline and those who dropped out by the follow-up survey regarding gender (t = −0.167, *p* > 0.05), age (t = −1.140, *p* > 0.05), personality (t = −0.816, *p* > 0.05), family social status (t = 0.597, *p* > 0.05), stress (t = −0.226, *p* > 0.05), approach goals (t = 0.832, *p* > 0.05), and avoidance goals (t = 0.409, *p* > 0.05). This indicates that the attrition did not significantly affect the results. Table 1 of descriptives for demographic variables is provided as follows:

### 2.2. Measures

Stress Perception: In this study, the stress subscale of the Depression Anxiety and Stress Scale-42 (DASS-42) was employed to measure stress among rural students. The DASS-42 is a widely employed 42-item self-report instrument designed to assess depression, anxiety, and stress [60]. The stress subscale comprises 14 items, each rated on a 4-point scale ranging from 0 to 3. Participants’ total scores on stress subscale ranged from 0 to 42, with higher scores indicating higher levels of stress. The stress subscale demonstrated a high degree of reliability, as evidenced by Cronbach’s alpha coefficients of 0.8842 at baseline and 0.8889 at follow-up.

Approach-Avoidance Achievement Goal Orientation: The present study utilized the Achievement Goal Questionnaire (AGQ), originally developed by Elliot and McGregor (2001) to evaluate achievement goals. The AGQ consists of 12 items, with six items assessing approach goals and six items assessing avoidance goals. Total scores range from 6 to 30, with higher scores indicating a more pronounced achievement goal orientation. The questionnaire was translated into Chinese to accommodate the linguistic preferences of the student population in China [61]. The instrument exhibited high reliability, with Cronbach’s alpha coefficients for approach goals at baseline and follow-up being 0.8117 and 0.8120, respectively, and for avoidance goals at baseline and follow-up being 0.6691 and 0.6561, respectively.

### 2.3. Data Analysis

In this study, data were analyzed using the statistical software Stata 15.0 and the structural equation modeling software Mplus 7.4. The initial phase of the analysis involved Stata to conduct descriptive statistics and correlation analyses regarding stress perception and achievement goal orientation. Subsequently, Mplus 7.4 was utilized to construct a cross-lagged model aimed at analyzing the predictive relationship between stress perception and achievement goal orientation. An optimal model fit was characterized by the following criteria: (1) comparative fit index (CFI) > 0.90 (superior fit ≥ 0.95), (2) Tucker-Lewis index (TLI) > 0.90 (superior fit ≥ 0.95), (3) root mean square error of approximation (RMSEA) < 0.10 (superior fit ≤ 0.06), and (4) standardized root mean square residual (SRMR) < 0.10.

## 3. Results

### 3.1. Descriptive Statistics and Correlation Analysis of Stress Perception and Achievement Goal Orientation

Table 2 presents the mean values, standard deviations, and correlation coefficients for stress perception and achievement goal orientation. Overall, the stress levels of rural students at college were within a normal range, decreasing from 12.114 in the junior year to 11.544 in the senior year. A paired-samples *t*-test revealed that the decline in stress levels was statistically significant (*p* < 0.05). Rural students’ approach goals were generally higher than their avoidance goals. Specifically, approach goals increased from 20.897 in the junior year to 20.910 in the senior year, while avoidance goals rose from 17.720 in the junior year to 17.869 in the senior year. Although there was an increase in the proportion of students with approach goals and those with avoidance goals, neither change was statistically significant (paired-samples *t*-test, *p* > 0.05). Correlation analysis indicated that at baseline, stress perception was significantly correlated exclusively with avoidance goals (r = 0.211, *p* < 0.05) and not with approach goals (r = 0.027, *p* > 0.05). At follow-up, a similar pattern was observed, with a significant correlation between stress perception and avoidance goals (r = 0.314, *p* < 0.05). Furthermore, stress perception at baseline was significantly correlated with avoidance goals at follow-up (r = 0.242, *p* < 0.05), and stress perception at follow-up was significantly correlated with the avoidance goals at baseline (r = 0.205, *p* < 0.05). In conclusion, stress perception, both concurrent and non-concurrent, was found to be associated only with avoidance goals.

### 3.2. Cross-Lagged Analysis between Stress Perception and Achievement Goal Orientation

**Figure 1:**
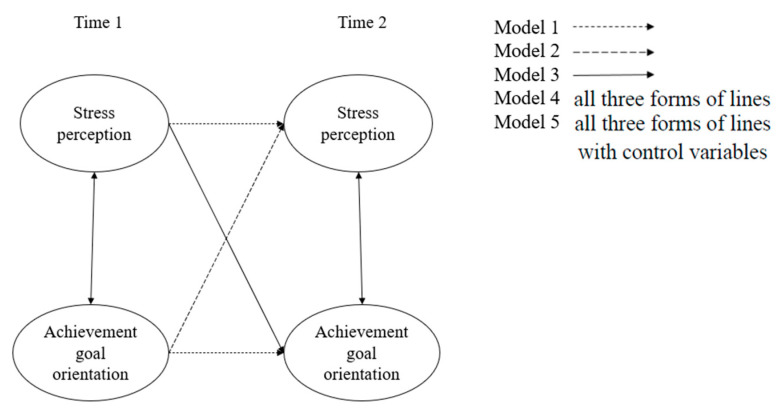
Cross-lagged models between stress perception and achievement goal orientation.

As illustrated in Figure 1, the following models were constructed to explore the relationship between stress perception and achievement goal orientation. Model 1 was the autoregressive model, which examined the stability of stress perception and achievement goal orientation. Model 2 built on Model 1 by including autoregressive paths and assessing the predictive effects of achievement goal orientation at baseline on stress perception at follow-up. Model 3 also included autoregressive paths but focused on the predictive effects of stress perception at baseline on achievement goal orientation at follow-up. Model 4 integrated the autoregressive and cross-lagged paths from Models 1, 2, and 3 to provide a comprehensive view of the relationships between the variables. Model 5 extended Model 4 by incorporating demographic variables, such as age, gender, personality, and family social status, to further refine the analysis.

Table 3 presents the model fit metrics for the stress perception and approach goals. Table 4 provides the autoregressive and the cross-lagged coefficients for these variables. The results of Table 3 indicate that Models 1–5 were well-fitted (CFI > 0.90, TLI > 0.90, RMSEA < 0.10, SRMR < 0.10). According to Table 4, autoregressive effects were significant (*p* < 0.05), suggesting that stress perception and approach goals remained stable from baseline to follow-up. However, approach goals did not significantly predict stress perception (β = −0.009 in Model 5, *p* > 0.05), and stress perception was not a significant predictor of approach goals (β = 0.065 in Model 5, *p* > 0.05). Additionally, the inclusions of the four control variables, namely age, gender, personality, and family social status, did not have a noticeable impact on the results.

Table 5 presents the model fit metrics for the stress perception and avoidance goals. Table 6 provides the autoregressive and the cross-lagged coefficients for relevant variables. Models 1–5 also fitted very well according to the results in Table 5 (CFI > 0.90, TLI > 0.90, RMSEA < 0.10, SRMR < 0.10). According to Table 6, the autoregressive coefficients from Model 1 show that stress perception (β = 0.641 in Model 1, *p* < 0.05) and avoidance goals (β = 0.526 in Model 1, *p* < 0.05) were stable from baseline to follow-up. Furthermore, the cross-lagged coefficients of Model 2–5 revealed that avoidance goals significantly predicted stress perception (β = 0.096 in Model 5, *p* < 0.05), and stress significantly predicted avoidance goals (β = 0.119 in Model 5, *p* < 0.05). The inclusion of control variables did not affect the bidirectional predictive relationship between avoidance goals and stress perception.

## 4. Discussion

The descriptive statistics revealed a significant decline in stress levels among Chinese rural college students from their junior to senior years, consistent with findings from a previous study [62]. This decline may be attributed to the reduction in academic stress during the fourth year of college. In Chinese universities, the curriculum is predominantly structured during the first 3 years [63]. In contrast, the fourth year provides more opportunities for students to prepare for further education or to seek employment. This reduction in academic demands likely contributes to the observed decrease in academic stress during the senior year. Nevertheless, the employment and interpersonal stress encountered in the fourth year remain largely consistent with that of the third year. Chinese students often begin engaging in employment-related activities as early as their third year, preparing for the workforce through internships and social practices [64], which inevitably heightens stress perceptions related to the workforce in the third year in advance and continues into the fourth year. Additionally, as students become more acquainted with faculty and peers, their interpersonal relationships tend to stabilize during their junior and senior years.

Achievement goal orientations among Chinese rural college students remained relatively stable from junior to senior year, consistent with previous research [65]. Temperament and personality are believed to influence achievement goals based on the hierarchical model of achievement motivation, with personality being a stronger predictor than temperament [66]. Given that personality traits change slowly over time, achieving significant short-term changes in these traits poses a considerable challenge.

The results of the cross-lagged model indicated no significant association between approach goals and stress perception among Chinese rural college students from junior to senior year. However, a clear positive reciprocal relationship was observed between avoidance goals and stress perception. These findings align with the hierarchical model of approach-avoidance motivation, which suggests that stress, as a negative emotion, is associated with avoidance goals but not with approach goals. Consequently, the two hypotheses are validated. In a meta-analysis of achievement goals and emotions, Huang [51] noted that while the correlation between performance avoidance goals and positive achievement emotions was weak, the correlation with negative achievement emotions was substantial. Similarly, mastery avoidance goals exhibited a small correlation with positive achievement emotions and a large correlation with negative achievement emotions. This suggests that both mastery and performance avoidance goals are more closely linked to negative emotions.

However, other studies involving Chinese university students have indicated that negative emotions, such as anxiety [61] and depression [49], are more likely to decrease approach goal orientation rather than increase avoidance goal orientation during the junior and senior years. This discrepancy may arise from differences in negative emotions. For instance, individuals with depression may lose motivation to avoid negative events, as they may perceive misfortunes as inevitable and evasion as futile [67]. Rural college students in China often exhibit distinct characteristics compared to their urban counterparts, as they come from economically disadvantaged and socially marginalized backgrounds. This background can lead to heightened emotional sensitivity and introversion [68], thereby influencing the relationship between negative emotions and achievement goals [69]. Therefore, future research should explore how diverse group characteristics and emotional states impact achievement goals.

Attention to rural college students’ academic performance and emotions should concentrate on breaking the vicious cycle of stress perception and avoidance goal orientation. Given the challenges in modifying achievement goals in the short term, educators should focus on alleviating stress experienced by rural college students through academic support—such as providing a supportive learning environment, teaching effective learning strategies, and promoting cooperative learning [70]; employment support—such as offering job shadowing opportunities, job training, and employment subsidies [71]; and interpersonal support—such as organizing exchange sessions and counseling [72].

## 5. Limitations

This study has several limitations. First, it relied on self-reported questionnaires to measure stress perception and achievement goals, which may not accurately capture the participants’ actual experiences. Although the Achievement Goals Questionnaire (AGQ) is widely used for assessing achievement goals, future research should consider using various measurement tools to assess potential differences and enhance the robustness of the findings. Second, the participants in this study were rural college students from public universities in Beijing. As the capital of China with numerous higher education institutions, students from colleges and universities at Beijing may be more academically gifted compared to their peers in other regions, which may affect the generalizability of the results. Future research should include a more diverse sample by incorporating students from different regions of China to address this issue. Third, the data presented in this study are based on two time points, which may also limit the generalizability of the findings. Additionally, the study focused solely on stress perception as a negative emotion and used a two-dimensional categorization of achievement goals. To provide a more comprehensive and nuanced understanding, future research could benefit from including a wider range of time periods and a broader set of variables.

## 6. Conclusions

Based on a two-wave random intercept cross-lagged model, the study analyzed the prospective relationship between the stress perception and approach-avoidance achievement goal orientation among Chinese rural students at college. First, there was a notable decline in stress levels among Chinese rural college students from their junior year to their senior year. Second, the achievement goal orientations of these students were relatively stable from junior to senior year while their approach goals scored higher than avoidance goals. Third, there was no significant association between approach goals and stress perception over the course of the study. However, a positive mutual predictive relationship was observed between avoidance goals and stress perception. It is noted that educators should focus on alleviating stress experienced by rural college students, such as academic support, employment assistance, and interpersonal support to prevent the vicious cycle of stress perception and avoidance goal orientation.

## Figures and Tables

**Table 1:** Descriptives of demographic variables.

Variables	Definitions	Mean	S.D.
Age	Measured in years	21.738	0.992
Gender	Male = 1, female = 0	0.619	0.486
Personality	Please rate your level of extroversion: 1 (introversion) to 9 (extroversion)	5.343	1.582
Family social status	Upper class = 1, upper-middle class = 2, middle class = 3, lower-middle class = 4, lower class = 5	3.655	0.863

**Table 2:** Descriptive statistics and correlation analysis of stress perception and achievement goal orientation.

Variables	1	2	3	4	5	6
1. Stress perception (T1)	1.000					
2. Approach goals (T1)	0.027	1.000				
3. Avoidance goals (T1)	0.211 *	0.306 *	1.000			
4. Stress perception (T2)	0.589 *	0.004	0.205 *	1.000		
5. Approach goals (T2)	0.082	0.575 *	0.141 *	0.073	1.000	
6. Avoidance goals (T2)	0.242 *	0.152 *	0.422 *	0.314 *	0.343 *	1.000
Mean	12.114	20.897	17.720	11.544	20.910	17.869
Standard deviation	7.170	4.862	4.351	7.205	4.607	4.198

Note: T1 refers to the measures at baseline, while T2 refers to the measures at follow-up. * *p* < 0.05.

**Table 3:** Fit indices for stress perception and approach goals.

Model	χ^2^	df	RMSEA (90% CI)	SRMR	CFI	TLI	Comparison	∆X^2^	*p*
Model 1	192.319	50	0.072 (0.062–0.083)	0.033	0.964	0.953			<0.05
Model 2	192.108	49	0.073 (0.063–0.084)	0.033	0.964	0.952	M1–M2	0.211	<0.05
Model 3	188.863	49	0.073 (0.062–0.084)	0.029	0.965	0.953	M1–M3	3.456	<0.05
Model 4	188.664	48	0.074 (0.063–0.085)	0.028	0.965	0.952	M1–M4	3.655	<0.05
Model 5	227.716	80	0.058 (0.050–0.067)	0.025	0.964	0.948	M1–M5	−35.397	<0.05

Note: χ^2^ = Chi-square; df = degree of freedom; RMSEA = root mean square error of approximation; SRMR = standardized root mean square residual; CFI = comparative fit index; TLI = Tucker-Lewis index.

**Table 4:** Cross-lagged analysis between stress perception and approach goals.

Model	Autoregressive Path	β	Cross-Lagged Path	β
M1	Stress perception (T1) → Stress perception (T2)	0.649 *		
	Approach goals (T1) → Approach goals (T2)	0.663 *		
M2	Stress perception (T1) → Stress perception (T2)	0.650 *	Approach goals (T1) → Stress perception (T2)	−0.018
	Approach goals (T1) → Approach goals (T2)	0.662 *		
M3	Stress perception (T1) → Stress perception (T2)	0.651 *	Stress perception (T1) → Approach goals (T2)	0.075
	Approach goals (T1) → Approach goals (T2)	0.660 *		
M4	Stress perception (T1) → Stress perception (T2)	0.652 *	Approach goals (T1) → Stress perception (T2)	−0.018
	Approach goals (T1) → Approach goals (T2)	0.659 *	Stress perception (T1) → Approach goals (T2)	0.075
M5	Stress perception (T1) → Stress perception (T2)	0.638 *	Approach goals (T1) → Stress perception (T2)	−0.009
	Approach goals (T1) → Approach goals (T2)	0.656 *	Stress perception (T1) → Approach goals (T2)	0.065

Note: β = standardized coefficient. * *p* < 0.05.

**Table 5:** Fit indices for stress perception and avoidance goals.

Model	χ^2^	df	RMSEA (90% CI)	SRMR	CFI	TLI	Comparison	∆X^2^	*p*
Model 1	191.084	50	0.072 (0.061–0.083)	0.052	0.955	0.941			<0.05
Model 2	185.537	49	0.072 (0.061–0.083)	0.046	0.957	0.942	M1–M2	5.547	<0.05
Model 3	181.536	49	0.071 (0.060–0.082)	0.040	0.958	0.943	M1–M3	9.548	<0.05
Model 4	176.636	48	0.070 (0.059–0.082)	0.035	0.959	0.944	M1–M4	14.448	<0.05
Model 5	231.406	80	0.059 (0.050–0.068)	0.033	0.953	0.933	M1–M5	−40.322	<0.05

Note: χ^2^ = Chi-square; df = degree of freedom; RMSEA = root mean square error of approximation; SRMR = standardized root mean square residual; CFI = comparative fit index; TLI = Tucker-Lewis index.

**Table 6:** Cross-lagged analysis between stress perception and avoidance goals.

Model	Autoregressive Path	β	Cross-Lagged Path	β
M1	Stress perception (T1) → Stress perception (T2)	0.641 *		
	Avoidance goals (T1) → Avoidance goals (T2)	0.526 *		
M2	Stress perception (T1) → Stress perception (T2)	0.610 *	Avoidance goals (T1) → Stress perception (T2)	0.104 *
	Avoidance goals (T1) → Avoidance goals (T2)	0.543 *		
M3	Stress perception (T1) → Stress perception (T2)	0.654 *	Stress perception (T1) → Avoidance goals (T2)	0.155 *
	Avoidance goals (T1) → Avoidance goals (T2)	0.475 *		
M4	Stress perception (T1) → Stress perception (T2)	0.628 *	Avoidance goals (T1) → Stress perception (T2)	0.097 *
	Avoidance goals (T1) → Avoidance goals (T2)	0.495 *	Stress perception (T1) → Avoidance goals (T2)	0.149 *
M5	Stress perception (T1) → Stress perception (T2)	0.614 *	Avoidance goals (T1) → Stress perception (T2)	0.096 *
	Avoidance goals (T1) → Avoidance goals (T2)	0.492 *	Stress perception (T1) → Avoidance goals (T2)	0.119 *

Note: β = standardized coefficient. * *p* < 0.05.

## Data Availability

The data that support the findings of this study are available from the corresponding author upon reasonable request.

## References

[1] Pedrelli P., Nyer M., Yeung A., Zulauf C., Wilens T. (2015). College students: Mental health problems and treatment considerations. Acad. Psychiatry.

[2] Fawzy M., Hamed S.A. (2017). Prevalence of psychological stress, depression and anxiety among medical students in Egypt. Psychiatry Res..

[3] Schlarb A.A., Claßen M., Grünwald J., Vögele C. (2017). Sleep disturbances and mental strain in university students: Results from an online survey in Luxembourg and Germany. Int. J. Ment. Health Syst..

[4] Reddy K.J., Menon K.R., Thattil A. (2018). Academic stress and its sources among university students. Biomed. Pharmacol. J..

[5] Fairbrother K., Warn J. (2003). Workplace dimensions, stress and job satisfaction. J. Manag. Psychol..

[6] Grubic N., Badovinac S., Johri A.M. (2020). Student mental health in the midst of the COVID-19 pandemic: A call for further research and immediate solutions. Int. J. Soc. Psychiatry.

[7] Buse B., Kadir Y. (2024). The Relationship between Job-Finding Anxiety and Academic Engagement: A Study on Undergraduate Students In Faculty of Sports Sciences. Gymnasium.

[8] Chen T., Peng L., Yin X., Jing B., Yang J., Cong G., Li G. (2020). A policy category analysis model for tourism promotion in China during the COVID-19 pandemic based on data mining and binary regression. Risk Manag. Healthc. Policy.

[9] Fu P., Jing B., Chen T., Xu C., Yang J., Cong G. (2021). Propagation model of panic buying under the sudden epidemic. Front. Public Health.

[10] Peng Y., Lv S.B., Low S.R., Bono S.A. (2024). The impact of employment stress on college students: Psychological well-being during COVID-19 pandemic in China. Curr. Psychol..

[11] Darling C.A., McWey L.M., Howard S.N., Olmstead S.B. (2007). College student stress: The influence of interpersonal relationships on sense of coherence. Stress Health J. Int. Soc. Investig. Stress.

[12] Kim E.Y. (2022). Academic stress, interpersonal relationships, and college life adaptation of nursing students who experienced COVID-19. J. Korean Appl. Sci. Technol..

[13] Cheng M.W., Xie Y., Lo S.K. (2023). Making the College Transition in China-Rural First-Generation College Students and Their Parents. Soc. Educ. Res..

[14] Meng H., Li J., Loerbroks A., Wu J., Chen H. (2013). Rural/urban background, depression and suicidal ideation in Chinese college students: A cross-sectional study. PLoS ONE.

[15] Law S., Liu P. (2008). Suicide in China: Unique demographic patterns and relationship to depressive disorder. Curr. Psychiatry Rep..

[16] Meng C., Kang Y. (2019). Rural youths admitted to elite universities:“Empathy” and destiny. Chin. Educ. Soc..

[17] Xu Y. (2022). Dongshi as reflexive habitus to understand Chinese rural students’ academic success. Camb. J. Educ..

[18] Li X. (2019). Unpacking the extraordinary academic success of rural students. Chin. Educ. Soc..

[19] Chen J. (2022). Hysteresis effects and emotional suffering: Chinese rural students’ first encounters with the urban university. Sociol. Res. Online.

[20] Dong J. (2012). A Case Study Of "habitus" And "field": The Chinese Rural Students On The University Campus. Probl. Educ. 21st Century.

[21] Pillay A.L., Ngcobo H.S. (2010). Sources of stress and support among rural-based first-year university students: An exploratory study. S. Afr. J. Psychol..

[22] Hussain R., Guppy M., Robertson S., Temple E. (2013). Physical and mental health perspectives of first year undergraduate rural university students. BMC Public Health.

[23] Benita M.S., Supriya M.V. (2016). Acculturative stress: Effect on academic performance of rural migrant students. Int. J. Bus. Excell..

[24] Pelletier J.E., Lytle L.A., Laska M.N. (2016). Stress, health risk behaviors, and weight status among community college students. Health Educ. Behav..

[25] Haidar S.A., De Vries N.K., Karavetian M., El-Rassi R. (2018). Stress, anxiety, and weight gain among university and college students: A systematic review. J. Acad. Nutr. Diet..

[26] Van Jaarsveld C.H., Fidler J.A., Steptoe A., Boniface D., Wardle J. (2009). Perceived stress and weight gain in adolescence: A longitudinal analysis. Obesity.

[27] Safarzaie H., Nastiezaie N., Jenaabadi H. (2017). The relationship of academic burnout and academic stress with academic self-efficacy among graduate students. New Educ. Rev..

[28] Hitches E., Woodcock S., Ehrich J. (2022). Building self-efficacy without letting stress knock it down: Stress and academic self-efficacy of university students. Int. J. Educ. Res. Open.

[29] Martin M.M., Cayanus J.L., Weber K., Goodboy A.K. (2006). College students’ stress and its impact on their motivation and communication with their instructors. Stress and Mental Health of College Students.

[30] Park J., Chung S., An H., Park S., Lee C., Kim S.Y., Lee J.D., Kim K.S. (2012). A structural model of stress, motivation, and academic performance in medical students. Psychiatry Investig..

[31] Macan T.H., Shahani C., Dipboye R.L., Phillips A.P. (1990). College students’ time management: Correlations with academic performance and stress. J. Educ. Psychol..

[32] Mirzaei T., Oskouie F., Rafii F. (2012). Nursing students’ time management, reducing stress and gaining satisfaction: A grounded theory study. Nurs. Health Sci..

[33] Maehr M.L. (1989). Thoughts about motivation. Res. Motiv. Educ..

[34] Dweck C.S. (1986). Motivational processes affecting learning. Am. Psychol..

[35] Nicholls J.G. (1984). Achievement motivation: Conceptions of ability, subjective experience, task choice, and performance. Psychol. Rev..

[36] Elliot A.J., McGregor H.A. (2001). A 2 × 2 achievement goal framework. J. Personal. Soc. Psychol..

[37] Elliot A.J., Murayama K., Pekrun R. (2011). A 3 × 2 achievement goal model. J. Educ. Psychol..

[38] Miksza P., Tan L., Dye C. (2016). Achievement motivation for band: A cross-cultural examination of the 2× 2 achievement goal motivation framework. Psychol. Music.

[39] Chia Liu W., Wang C.J., Tan O.S., Ee J., Koh C. (2009). Understanding students’ motivation in project work: A 2× 2 achievement goal approach. Br. J. Educ. Psychol..

[40] Conroy D.E., Elliot A.J., Hofer S.M. (2003). A 2 × 2 achievement goals questionnaire for sport: Evidence for factorial invariance, temporal stability, and external validity. J. Sport Exerc. Psychol..

[41] Mascret N., Elliot A.J., Cury F. (2015). Extending the 3 × 2 achievement goal model to the sport domain: The 3 × 2 Achievement Goal Questionnaire for Sport. Psychol. Sport Exerc..

[42] Wu C.C. (2012). The cross-cultural examination of 3× 2 achievement goal model in Taiwan. Procedia-Soc. Behav. Sci..

[43] Elliot A.J., Harackiewicz J.M. (1996). Approach and avoidance achievement goals and intrinsic motivation: A mediational analysis. J. Personal. Soc. Psychol..

[44] McClelland D.C. (1951). Measuring motivation in phantasy: The achievement motive. Groups, Leadership and Men: Research in Human Relations.

[45] Linnenbrink E.A. (2005). The dilemma of performance-approach goals: The use of multiple goal contexts to promote students’ motivation and learning. J. Educ. Psychol..

[46] Huang C. (2016). Achievement goals and self-efficacy: A meta-analysis. Educ. Res. Rev..

[47] Heimerdinger S.R., Hinsz V.B. (2008). Failure avoidance motivation in a goal-setting situation. Hum. Perform..

[48] Świątkowski W., Dompnier B. (2021). When pursuing bad goals for good reasons makes it even worse: A social value approach to performance-avoidance goal pursuit. Soc. Psychol. Educ..

[49] Gao W., Ji J., Zhang W., Liu X. (2024). Depression and approach-avoidance achievement goals of Chinese undergraduate students: A four-wave longitudinal study. Br. J. Educ. Psychol..

[50] Elliot A.J. (2006). The hierarchical model of approach-avoidance motivation. Motiv. Emot..

[51] Huang C. (2011). Achievement goals and achievement emotions: A meta-analysis. Educ. Psychol. Rev..

[52] Roth S., Cohen L.J. (1986). Approach, avoidance, and coping with stress. Am. Psychol..

[53] Grigorescu D. (2020). Curiosity, intrinsic motivation and the pleasure of knowledge. J. Educ. Sci. Psychol..

[54] Lin Y., Wang J., Liu W., Jia Y. (2022). More Positive Emotion, Less Stress Perception?. Psychol. Res. Behav. Manag..

[55] Suls J., Fletcher B. (1985). The relative efficacy of avoidant and nonavoidant coping strategies: A meta-analysis. Health Psychol..

[56] Thwaites R., Freeston M.H. (2005). Safety-seeking behaviours: Fact or function? How can we clinically differentiate between safety behaviours and adaptive coping strategies across anxiety disorders?. Behav. Cogn. Psychother..

[57] Sun J., Dunne M.P., Hou X.Y., Xu A.Q. (2013). Educational stress among Chinese adolescents: Individual, family, school and peer influences. Educ. Rev..

[58] McGregor H.A., Elliot A.J. (2002). Achievement goals as predictors of achievement-relevant processes prior to task engagement. J. Educ. Psychol..

[59] Liu X., Yuan Y., Gao W., Luo Y. (2024). Longitudinal trajectories of self-esteem, related predictors, and impact on depression among students over a four-year period at college in China. Humanit. Soc. Sci. Commun..

[60] Liu X., Zhu C., Dong Z., Luo Y. (2024). The Relationship between Stress and Academic Self-Efficacy among Students at Elite Colleges: A Longitudinal Analysis. Behav. Sci..

[61] Liu X., Zhang Y., Cao X., Gao W. (2024). Does anxiety consistently affect the achievement goals of college students? A four-wave longitudinal investigation from China. Curr. Psychol..

[62] Liu X., Li Y., Cao X. (2024). Bidirectional reduction effects of perceived stress and general self-efficacy among college students: A cross-lagged study. Humanit. Soc. Sci. Commun..

[63] D. Clemes M., A. Cohen D., Wang Y. (2013). Understanding Chinese university students’ experiences: An empirical analysis. Asia Pac. J. Mark. Logist..

[64] Yang J., Liu Z. (2020). A case study of the correlation between internship selection and graduation intention. Adv. Dev. Educ. Psychol..

[65] Lee Y.K., Wormington S.V., Linnenbrink-Garcia L., Roseth C.J. (2017). A short-term longitudinal study of stability and change in achievement goal profiles. Learn. Individ. Differ..

[66] Chen C., Zhang L.F. (2011). Temperament, personality and achievement goals among Chinese adolescent students. Educ. Psychol..

[67] Dickson J., MacLeod A. (2004). Brief report anxiety, depression and approach and avoidance goals. Cogn. Emot..

[68] Chen X., Wang L., Cao R. (2011). Shyness-sensitivity and unsociability in rural Chinese children: Relations with social, school, and psychological adjustment. Child Dev..

[69] Liu X., Zhang Y., Cao X. (2023). Achievement goal orientations in college students: Longitudinal trajectories, related factors, and effects on academic performance. Eur. J. Psychol. Educ..

[70] Hendriksen S.I., Yang L., Love B., Hall M.C. (2005). Assessing academic support: The effects of tutoring on student learning outcomes. J. Coll. Read. Learn..

[71] Arnold W.W. (2018). Strengthening College Support Services to Improve Student Transitioning to Careers. J. Coll. Teach. Learn..

[72] Grant-Vallone E., Reid K., Umali C., Pohlert E. (2003). An analysis of the effects of self-esteem, social support, and participation in student support services on students’ adjustment and commitment to college. J. Coll. Stud. Retent. Res. Theory Pract..

